# Pancreatic ^18^F-FDG uptake is increased in type 2 diabetes patients compared to non-diabetic controls

**DOI:** 10.1371/journal.pone.0213202

**Published:** 2019-03-19

**Authors:** Guido J. Bakker, Manon C. Vanbellinghen, Torsten P. Scheithauer, C. Bruce Verchere, Erik S. Stroes, Nyanza K. L. M. Timmers, Hilde Herrema, Max Nieuwdorp, Hein J. Verberne, Daniël H. van Raalte

**Affiliations:** 1 Department of Vascular Medicine, Amsterdam University Medical Centers, Amsterdam, The Netherlands; 2 Department of Experimental Vascular Medicine, Amsterdam University Medical Centers, Amsterdam, The Netherlands; 3 Diabetes Center, Department of Internal Medicine, Amsterdam University Medical Centers, Amsterdam, The Netherlands; 4 Department of Surgery and Department of Pathology and Laboratory Medicine, BC Children’s Hospital, University of British Columbia, Vancouver, British Columbia, Canada; 5 Wallenberg Laboratory, Department of Molecular and Clinical Medicine, Sahlgrenska Academy, University of Gothenburg, Gothenburg, Sweden; 6 ICaR, Amsterdam University Medical Centers, Amsterdam, The Netherlands; 7 Department of Radiology and Nuclear Medicine, Amsterdam University Medical Centers, Amsterdam, The Netherlands; Mahidol University, THAILAND

## Abstract

**Introduction:**

Increasing evidence indicates that the development of type 2 diabetes is driven by chronic low grade beta-cell inflammation. However, it is unclear whether pancreatic inflammation can be noninvasively visualized in type 2 diabetes patients. We aimed to assess pancreatic ^18^F-FDG uptake in type 2 diabetes patients and controls using ^18^F-fluorodeoxylglucose positron emission tomography/computed tomography (^18^F-FDG PET/CT).

**Material and methods:**

In this retrospective cross-sectional study, we enrolled 20 type 2 diabetes patients and 65 controls who had undergone a diagnostic ^18^F-FDG PET/CT scan and obtained standardized uptake values (SUVs) of pancreas and muscle. Pancreatic SUV was adjusted for background uptake in muscle and for fasting blood glucose concentrations.

**Results:**

The maximum pancreatic SUVs adjusted for background muscle uptake (SUV_max.m_) and fasting blood glucose concentration (SUV_glucose_) were significantly higher in diabetes patients compared to controls (median 2.86 [IQR 2.24–4.36] compared to 2.15 [IQR 1.51–2.83], p = 0.006 and median 2.76 [IQR 1.18–4.34] compared to 1.91 [IQR 1.27–2.55], p<0.001, respectively). In linear regression adjusting for age and body mass index, diabetes remained the main predictor of SUV_max.m_ and SUV_glucose_.

**Conclusion:**

Pancreatic ^18^F-FDG uptake adjusted for background muscle uptake and fasting blood glucose concentration was significantly increased in type 2 diabetes patients.

## Introduction

Hyperglycemia and type 2 diabetes (T2D) are driven by a decline in beta-cell function and mass against a background of insulin resistance [1,2]. Beta-cell dysfunction is present before diabetes onset [3,4] and worsens over time, determining the progressive course of the disease [5,6]. The underlying pathophysiological mechanisms of beta-cell dysfunction are yet to be unraveled. However, increasing evidence indicates that chronic low-grade Langerhans islet inflammation is involved. Several studies have linked increased presence and a pro-inflammatory phenotype of islet macrophages to beta-cell inflammation, dysfunction and apoptosis, likely mediated through secretion of pro-inflammatory cytokines such as interleukin-1β and tumor necrosis factor-α [7–13]. In keeping with this idea, various anti-inflammatory therapies have been shown to modestly improve beta-cell function in type 2 diabetes patients [14]. Taken together, these lines of evidence suggest a role for islet inflammation in the development of type 2 diabetes.

The difficulty in obtaining human pancreatic tissue combined with the limitations of post-mortem assessments, islet cultures and rodent studies, has hampered the study of islet inflammation in diabetes [15]. Thus, noninvasive visualization of pancreatic inflammation would greatly increase insight into the pathogenesis of type 2 diabetes and aid to assess effects of beta-cell-sparing interventions. A well accepted imaging modality for visualization of inflammation is positron emission tomography/computed tomography (PET/CT) with ^18^F-fluorodeoxylglucose (^18^F-FDG) [16]. This technique relies on the fact that activated inflammatory cells have increased glucose metabolism and thereby higher ^18^F-FDG uptake than normal cells [17]. For example, it was shown that FDG uptake in carotid arerial wall of diabetes subjects was increased compared to controls [18]. Moreover, ^18^F-FDG PET/CT scanning of plaque inflammation has been used to assess efficacy of anti-inflammatory therapy [19]. Here we hypothesized that compared to non-diabetic controls, type 2 diabetes patients have increased pancreatic ^18^F-FDG uptake on PET/CTs.

## Materials and methods

### Study design and patient selection

In this retrospective study, we included patients referred to the Department of Nuclear Medicine of our hospital for a ^18^F-FDG PET/CT between January 1^st^ and July 31^st^, 2015. The following data were extracted from electronic medical files: age, sex, height, weight, indication for ^18^F-FDG PET/CT, medical history, confirmation of diabetes diagnosis according to ADA criteria, diabetes duration, use of medication and fasting blood glucose concentrations. For this study, a waiver from the local ethical committee (Medisch Ethische Toetsings Commissie AMC) was obtained that stated that no informed consent was needed and data did not have to be fully anonymized, as the data were collected as part of a routine procedure and were not used to guide treatment decisions or influenced planned treatment. Patients were excluded if they met one of the following criteria: age <18 years; fasting blood glucose concentration ≥7.0 mmol/L on the day of the PET/CT without a medical history of diabetes; acute or chronic pancreatitis or the presence of pancreatic calcifications or cystic lesions; intra-abdominal malignancy; type 1 diabetes; HIV infection; use of oral or i.v. antibiotics in the 6 weeks preceding the PET/CT; use of systemic immunomodulatory drugs in the 2 weeks preceding the PET/CT; radiotherapy or chemotherapy in the 6 weeks preceding the PET/CT; alcohol abuse, defined as an intake of 5 or more units daily; use of recreational drugs; imaging that did not include the whole pancreas; only low-dose PET/CT available; insufficient imaging or; insufficient available patient data.

### ^18^F-FDG PET/CT acquisition and analysis

^18^F-FDG PET/CTs were acquired using a Philips Gemini TF-16 PET/CT scanner (Philips Medical Systems, Eindhoven, the Netherlands). All scans were performed according to the local ^18^F-FDG PET/CT scanning protocol (see Supporting Information). Prior to ^18^F-FDG administration, fasting capillary blood glucose concentrations were measured with a blood glucose meter (StatStrip, Nova Biomedical Corporation, Waltham, MA, USA). Dosages of ^18^F-FDG ranged from 180 to 400 MBq depending on BMI (180 MBq for BMI <28 kg/m^2^; 240MBq for BMI 28–35 kg/m^2^; 300 MBq for BMI 35–40 kg/m^2^ and 400MBq for BMI >40 kg/m^2^). PET/CTs were performed 60–90 min after injection of ^18^F-FDG.

^18^F-FDG PET/CT images were analyzed using Hybrid Viewer (Hermes Medical Solutions, Stockholm, Sweden). PET images were reconstructed iteratively using ordered-subset expectation maximization software. PET, CT, and fused PET/CT images were available for review and were displayed as non-corrected and attenuation-corrected images in axial, coronal, and sagittal planes. Regions of interest (ROIs) were manually drawn over the tissues of interest on axial views on the CT images on a slice-by-slice basis, so as to cover the entire volume of the pancreas (S1 Fig) or spleen. A circular ROI was also drawn on four consecutive transverse slices of the erector spinae muscle in order to be able to adjust for background uptake of ^18^F-FDG. Separate ROIs were merged into a volume of interest. Mean standardized uptake values (SUV_mean_) and SUV of the hottest voxel (SUV_max_) within the defined regions were automatically generated by the software. The assessor was blinded to the patients’ diabetes status. A detailed description of PET/CT acquisition and analysis can be found in the Supporting Information.

### Study outcomes

The main outcome was the difference between diabetes patients and controls in SUV_max.m_, the maximum pancreatic SUV corrected for background uptake in muscle according to the following formula:

$${SUV}_{max.m}=\frac{{SUV}_{max\;pancreas}-{SUV}_{mean\;muscle}}{{SUV}_{mean\;muscle}}$$

As secondary outcomes, we determined SUV_glucose_, the maximum pancreatic SUV corrected for blood glucose concentration, according to the following formula [20].

$${SUV}_{glucose}={SUV}_{max\;pancreas}\times \frac{Fasting\;blood\;glucose}{7}$$

As a sensitivity analysis, we repeated all analyses using spleen as background:

$${SUV}_{max.s}=\frac{{SUV}_{max\;pancreas}-{SUV}_{mean\;spleen}}{{SUV}_{mean\;spleen}}$$

### Statistical analyses

Data are shown as mean with standard deviation (SD) or median with interquartile range (IQR). Differences between the diabetes patients and controls were assessed using Pearson’s chi-square test for proportions and the student’s t-test or the Mann-Whitney U test for normally and non-normally distributed data, respectively. Spearman’s correlation was used to assess correlations between parameters of interest. Multivariate linear regression analysis was performed to further assess the relationship between SUV_max.m_, SUV_glucose_ and SUV_max.s_ and correlating variables. We verified whether the assumptions of linear regression model were met, including normality, linearity, homoscedasticity, and non-collinearity. Statistical analyses were performed using SPSS Statistics software, version 24 (IBM, Armonk, New York). *P*-values <0.05 were considered statistically significant.

## Results

### Patients

Between January 1^st^ and July 31^st^ of 2015, 802 patients were referred for an ^18^F-FDG PET/CT. After application of the exclusion criteria, 85 subjects were included in the analysis (S2 Fig). Forty-eight patients (56.5%) underwent ^18^F-FDG PET/CT scanning for primary tumor staging, 18 (21.2%) for diagnosis of malignancy, 16 (18.8%) for follow-up of malignancy and 3 (3.5%) for evaluation of suspected inflammation. Baseline characteristics are summarized in Table 1. Of the 85 participants, 20 (23.5%) had T2D and 65 (76.5%) were non-diabetic. There was no significant difference in mean age or sex distribution between the groups. BMI and fasting glucose were significantly higher in the diabetes patients compared to controls.

**Table 1:** Baseline characteristics.

Characteristics	Diabetes patients n = 20	Controls n = 65	*p*
Age in years	60.6 (10.7)	55.1 (14.4)	0.122
Male sex, n (%)	5 (25)	19 (29.2)	0.713
Weight in kg	82.9 (22.3)	72.4 (13.1)	0.056
BMI in kg/m^2^	29.1 (6.7)	25.1 (4.7)	0.004
Glucose in mmol/L	7.2 (1.6)	5.3 (0.7)	<0.001
Diabetes duration in years	7.2 (6.7)	-	-
Metformin use, n (%)	17 (85)	-	-
Insulin use, n (%)	3 (15)	-	-

n, number of patients. Data are mean (standard deviation) unless specified otherwise.

### Pancreatic SUVs on ^18^F-FDG PET/CT

**Fig 1:**
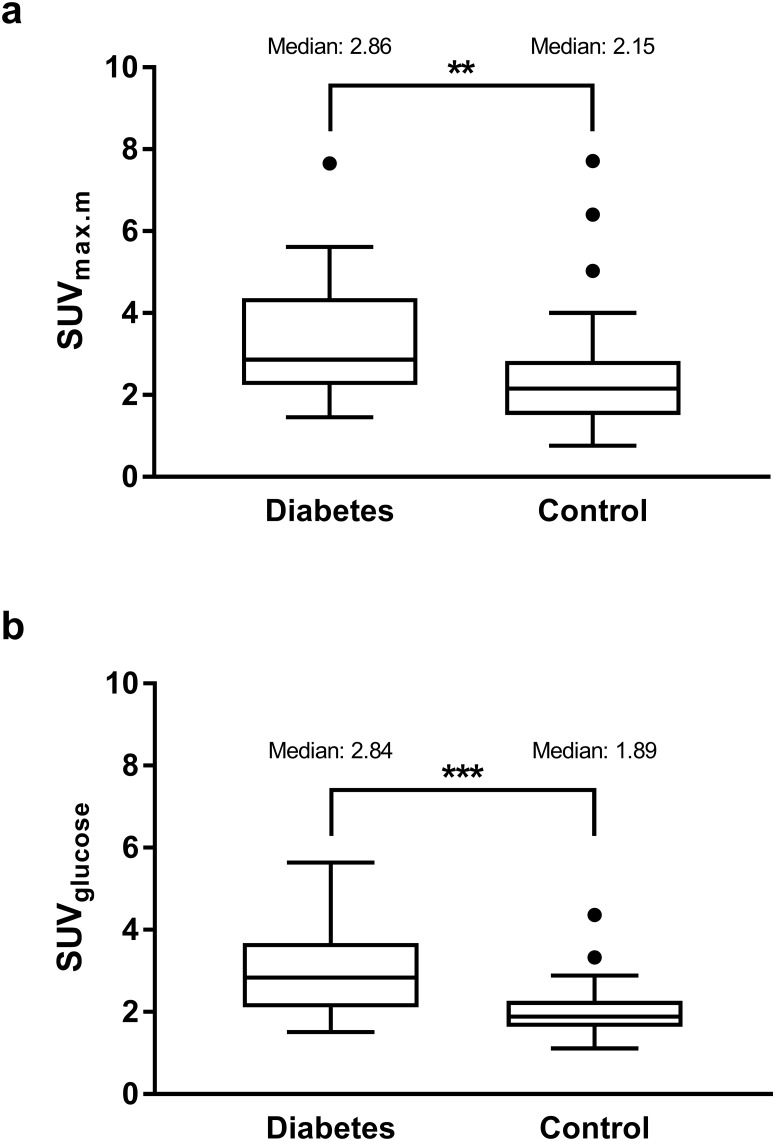
Pancreatic glucose uptake. Maximum pancreatic SUV corrected for background muscle uptake (a) and for fasting blood glucose (b). Shown are median values with IQR. **, *p*<0.01; ***, *p*<0.001.

SUV_max.m_ and SUV_glucose_ were significantly higher in T2D patients compared to controls: median (IQR) 2.86 (2.24–4.36) versus 2.15 (1.52–2.83), *p* = 0.006, for SUV_max.m_ and median (IQR) 2.84 (2.11–3.68) versus 1.89 (1.64–2.27), *p*<0.001, for SUV_glucose_ (Fig 1 and S1 Table). SUV_max.m_ was positively correlated with diabetes presence (*r*_*s*_ = 0.30, *p* = 0.005) and BMI (*r*_*s*_ = 0.26, *p* = 0.016) and negatively correlated with age (*r*_*s*_ = -0.23, *p* = 0.036). SUV_glucose_ was correlated with diabetes presence (*r*_*s*_ = 0.43, *p*<0.001) and BMI (*r*_*s*_ = 0.39, *p<* 0.001), but not with age (*r*_*s*_ = -0.06, *p* = 0.616).

In simple linear regression, diabetes patients had a 0.94 (95%CI 0.28–1.61) higher SUV_max.m_ and a 1.06 (95%CI 0.68–1.45) higher SUV_glucose_ compared to controls, respectively. After adjustment for BMI and age, diabetes remained a significant predictor of both outcomes with a 0.93 (95%CI 0.26–1.61) higher SUV_max.m_ and a 1.00 (95%CI 0.59–1.42) higher SUV_glucose_ compared to controls, respectively (S1 Table). In a sensitivity analysis using spleen as background, the presence of diabetes remained a significant predictor (S1 Table). We found no significant interaction between diabetes presence and BMI (*p* = 0.872) or age (*p* = 0.131).

## Discussion

To our knowledge, this is the first study to show that type 2 diabetes patients have significantly increased background-corrected pancreatic ^18^F-FDG uptake compared to non-diabetic controls. This association persisted after adjustment for BMI and age as well as after correction for prevailing glucose concentrations. Our study is in line with previous evidence that suggest an important role for beta-cell inflammation in T2D development. In addition, this study suggests that pancreatic inflammation—possibly reflecting islet inflammation—can be imaged *in vivo* and paves the way for prospective studies that investigate the role of inflammation on beta-cell function and diabetes development over time and modulating effects of anti-inflammatory therapies.

As beta-cell function starts to decline years before the clinical diagnosis of T2D [21], islet inflammation may be most prominent in early diabetes. Considering the patients in our study had on average been diagnosed with diabetes for approximately 7 years and that diabetes onset occurs 4–8 years before clinical diagnosis [22], it is possible that a study in patients with a more recent-onset diabetes would reveal an even greater difference between diabetes patients and controls. However, the relatively small sample size of 20 diabetes patients in our study precluded such an analysis.

Previously, Honka *et al*. assessed pancreatic ^18^F-FDG uptake in 25 morbidly obese subjects and found no difference between patients with and without T2D [23]. However, in that study ^18^F-FDG uptake was not adjusted for background uptake nor fasting plasma glucose concentrations, despite the inverse correlation between ^18^F-FDG uptake and fasting plasma glucose concentrations that was reported. Moreover, the patients included in the study of Honka *et al*. had severe pancreatic steatosis, raising the possibility that ^18^F-FDG uptake was mainly mediated by adipocytes instead of islets.

Our retrospective study has several limitations. First, even though ^18^F-FDG PET/CT is widely used to image inflammation, glucose uptake could also be indicative of metabolic activity rather than inflammation. As such, beta cells stressed by chronic hyperglycemia could have higher metabolic activity. Thus, even though islets of T2D patients largely fail the capacity to meet the increased insulin demand, this technique may not be specific enough to distinguish inflammation. Second, destruction of beta cells could lead to further decreased metabolic activity and lower ^18^F-FDG uptake compared to controls, resulting in an underestimation of the PET/CT signal in T2D subjects. Unfortunately, we did not have information on plasma insulin concentrations as a marker of beta cell activity. Thirdly, the Langerhans islet only take up a small portion of the pancreas. As such, the PET/CT signal within the pancreas may reflect exocrine pancreatic tissue. Nevertheless, in a non-obese diabetic mouse model FDG uptake was shown to be 2–3 times higher in islets that were infiltrated by immune cells than in remaining pancreas [24]. Thus, assuming several islets within one voxel may be infiltrated, the increased maximum ^18^F-FDG signal in the diabetes subjects is in line with previous data. Finally, most participants in this study underwent PET-CT scanning for diagnosis or follow-up of malignancy. Although indications for scanning in the control group were similar to the diabetes group and patients with pancreatitis or intra-abdominal malignancies were excluded from the study, we cannot rule out that the state of inflammation in a diabetes population without (suspected) malignancy may be different.

In order to overcome the limitations of our study design, we used various methods to analyze pancreatic ^18^F-FDG uptake. First, we corrected pancreatic ^18^F-FDG uptake to background muscle uptake. Previously, increases in plasma glucose levels were associated with a significant increase in muscle uptake of ^18^F-FDG, despite the fact that endogenous glucose can competitively inhibit ^18^F-FDG uptake [25–28]. Thus, even though we feel that correction for background glucose uptake is necessary, correcting for ^18^F-FDG uptake in muscle may have led to an underestimation of background-corrected pancreatic signal in the diabetes patients. After using muscle as background tissue, we performed a sensitivity analysis using spleen as background and found similar results. This suggests that the impact of possible confounding factors is most likely limited. Finally, we used multivariate regression analysis to adjust for age and BMI, as age was significantly correlated with SUV_max.m_ and BMI was significantly different between the groups. Nevertheless, our findings should be confirmed in a prospective setting.

In conclusion, we showed that pancreatic ^18^F-FDG uptake, when adjusted for background uptake, is increased in type 2 diabetes patients, independent of BMI and age. Moreover, we showed that ^18^F-FDG PET/CT might be a viable tool for *in vivo* visualization of pancreatic inflammation in diabetes. The possibility of *in vivo* imaging of pancreatic inflammation offers a promising novel way to gain more insight in the processes underlying beta cell dysfunction in type 2 diabetes in prospective and intervention studies.

## Supporting information

S1 Fig ^18^F-FDG PET/CT images. Examples of CT (a), ^18^F-FDG PET/CT (b) and ^18^F-FDG PET (c) images from the abdominal region of a 44-year-old female with type 2 diabetes. The pancreas is encircled in all three images.
(TIF)

S2 Fig Flowchart of the study. (TIF)

S1 Table Linear regression analysis. (DOCX)

S1 Data Database containing raw data of the study. (SAV)
