# Supplementary figures and images for: Pancreatic 18F-FDG uptake is increased in type 2 diabetes patients compared to non-diabetic controls
Source: PLoS One. 2019 Mar 19;14(3):e0213202. doi: 10.1371/journal.pone.0213202 (PMC6424390; doi:10.1371/journal.pone.0213202)

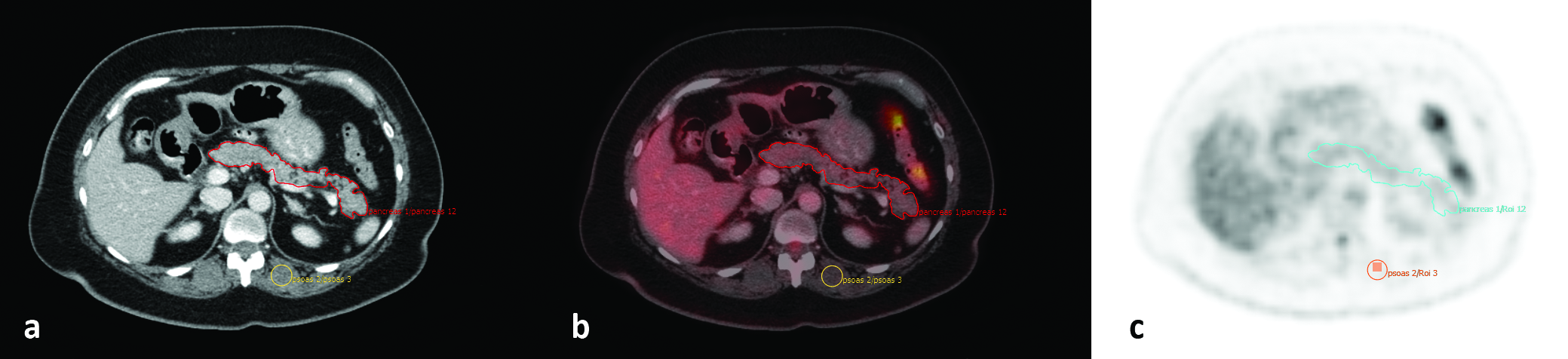

Supplement: S1 Fig — Examples of CT (a), 18F-FDG PET/CT (b) and 18F-FDG PET (c) images from the abdominal region of a 44-year-old female with type 2 diabetes. The pancreas is encircled in all three images. (TIF) [file pone.0213202.s001.tif]
