# Supplementary material for: Pancreatic 18F-FDG uptake is increased in type 2 diabetes patients compared to non-diabetic controls
Source: PLoS One. 2019 Mar 19;14(3):e0213202. doi: 10.1371/journal.pone.0213202 (PMC6424390; doi:10.1371/journal.pone.0213202)
Supplement: S1 Table — (DOCX) [file pone.0213202.s003.docx]

**Supporting information**

**PET/CT scanning protocol**

Patients were instructed to drink 2 liters of water and to not perform strenuous physical activities in the 24 hours preceding the scan. Patients were fasted for at least 6 hours except for glucose-free oral hydration before intravenous administration of ^18^F-FDG. Diabetic patients who used insulin were instructed not to use morning insulin if the PET/CT was to be performed in the morning. If the PET/CT was scheduled in the afternoon, they were instructed to use half their normal morning dose after a light breakfast and before the start of the fasting period. Patients who used oral glucose-lowering drugs were instructed to take the medication after the ^18^F-FDG PET/CT was acquired. If the indication for the ^18^F-FDG PET/CT was evaluation of inflammation, patients were asked to follow a low carbohydrate diet 24 hours prior to the scan. Prior to ^18^F-FDG administration, fasting capillary blood glucose concentrations were measured with a blood glucose meter (StatStrip, Nova Biomedical Corporation, Waltham, MA, USA). Oral contrast was used in all patients and consisted of a solution of 50 ml of the radiocontrast agent ioxithalamate (Telebrix™; Guerbet, Paris, France) and 800 ml water. This solution was given at two separate moments, one glass just before injection of the ^18^F-FDG bolus and the rest immediately afterwards. For the injections of ^18^F-FDG, a catheter was inserted into the antecubital vein. Dosages of ^18^F-FDG ranged from 180 to 400 MBq depending on BMI (180 MBq for BMI <28 kg/m^2^; 240MBq for BMI 28-35 kg/m^2^; 300 MBq for BMI 35-40 kg/m^2^ and 400MBq for BMI >40 kg/m^2^). PET/CTs were performed 60-90 min after injection of ^18^F-FDG.

**^18^F-FDG PET/CT acquisition and analysis**

^18^F-FDG PET/CTs were acquired using a Philips Gemini TF-16 PET/CT scanner (Philips Medical Systems, Eindhoven, the Netherlands). First, a CT using intravenous contrast (porto-venous phase) was acquired from the base of the skull to the thighs to be used for anatomical localization and attenuation correction. The scanning parameters were: 120 kVp, 50 mA/slice, rotation time 0.75 seconds, and slice thickness/interval 3.0 mm. Directly after CT acquisition, three-dimensional emission scans were obtained of the same region over 10 bed positions with a scanning time of 2 minutes per bed position and 50% overlap between 2 bed positions. For both CT and PET imaging the patient was in supine position with the arms above the head and allowed to breathe freely.

^18^F-FDG PET/CT images were analyzed using Hybrid Viewer^TM^ (Hermes Medical Solutions, Stockholm, Sweden). PET images were reconstructed iteratively using ordered-subset expectation maximization software. PET, CT, and fused PET/CT images were available for review and were displayed as non-corrected and attenuation-corrected images in axial, coronal, and sagittal planes. Regions of interest (ROIs) were manually drawn over the pancreas or spleen on axial views in each patient with the aid of corresponding CT images on a slice-by-slice basis, so as to cover the entire volume of the pancreas and spleen. A circular region of interest was also drawn on four consecutive transverse slices of the erector spinae muscle in order to be able to adjust for background uptake of ^18^F-FDG. Separate ROIs were merged to form a volume of interest (VOI). Mean standardized uptake values (SUV_mean_) and SUV of the hottest voxel (SUV_max_) within the defined VOIs were automatically generated by the viewing software. The assessor was blinded to the patients’ diabetes status.

**Supporting Table**

**S1 Table. Linear regression analysis.**

|  | **SUV_max.m_** | | |  | **SUV_glcuose_** | | |  | **SUV_max.s_** | | |  |
| --- | --- | --- | --- | --- | --- | --- | --- | --- | --- | --- | --- | --- |
| **Variable** | **B** | **95%CI** | ***p*-value** |  | **B** | **95%CI** | ***p*-value** |  | **B** | **95%CI** | ***p*-value** |  |
| Constant | 2.88 | 1.12 – 4.64 | 0.002 |  | 1.70 | 0.63 – 2.77 | 0.002 |  | 0.79 | 0.07 – 1.50 | 0.032 |  |
| Diabetes | 0.93 | 0.26 – 1.61 | 0.008 |  | 1.00 | 0.59 – 1.42 | <0.001 |  | 0.28 | 0.00 – 0.56 | 0.047 |  |
| BMI | 0.04 | -0.01 – 0.09 | 0.130 |  | 0.02 | -0.01 – 0.05 | 0.244 |  | -0.002 | -0.02 – 0.02 | 0.884 |  |
| Age | -0.03 | -0.05 – -0.01 | 0.008 |  | -0.003 | -0.02 – 0.01 | 0.624 |  | -0.004 | -0.01 – 0.00 | 0.324 |  |
| BMI, body mass index; SUV_glucose_, maximum pancreatic SUV corrected for blood glucose concentration; SUV_max.m_, maximum pancreatic SUV corrected for background muscle uptake; SUV_max.s_, maximum pancreatic SUV corrected for background spleen uptake. | | | | | | | | | | | | |
